# Oncolytic Viruses in the Treatment of Bladder Cancer

**DOI:** 10.1155/2012/404581

**Published:** 2012-07-29

**Authors:** Kyle G. Potts, Mary M. Hitt, Ronald B. Moore

**Affiliations:** ^1^Department of Oncology, Faculty of Medicine and Dentistry, University of Alberta, Edmonton, AB, Canada T6G 2E1; ^2^Department of Surgery, Faculty of Medicine and Dentistry, University of Alberta, Edmonton, AB, Canada T6G 2B7

## Abstract

Bladder carcinoma is the second most common malignancy of the urinary tract. Up to 85% of patients with bladder cancer are diagnosed with a tumor that is limited to the bladder mucosa (Ta, T1, and CIS). These stages are commonly termed as non-muscle-invasive bladder cancer (NMIBC). Although the treatment of NMIBC has greatly improved in recent years, there is a need for additional therapies when patients fail bacillus Calmette-Guérin (BCG) and chemotherapeutic agents. We propose that bladder cancer may be an ideal target for oncolytic viruses engineered to selectively replicate in and lyse tumor cells leaving normal cells unharmed. In support of this hypothesis, here we review current treatment strategies for bladder cancer and their shortcomings, as well as recent advancements in oncolytic viral therapy demonstrating encouraging safety profiles and antitumor activity.

## 1. Transitional Cell Carcinomas

In the United States, it is estimated that 73,510 men and women (55,600 men and 17,910 women) will be diagnosed with and 14,880 will die of *cancer of the urinary bladder* in 2012, making it the fourth and ninth most common cancers among men and women, respectively [1]. The most common cause for bladder cancer is smoking and other toxin exposure (i.e., petrochemical industry), where the carcinogen is removed from the body by the kidney and stored for long periods of time in the bladder. This results in destabilization of the urothelium resulting in a field effect.

More than 90% of cancers in the bladder are transitional cell carcinomas (TCCs), which have more recently been termed urothelial cell carcinomas [2]. Approximately, 80% of patients with bladder cancer have tumors that are limited to the mucosa of the bladder (stage Ta and carcinoma *in situ *(CIS)) or penetrate into the submucosa (stage T1) [3, 4]. These superficial bladder cancers are now being described as non-muscle-invasive bladder cancer (NMIBC) (Reviewed in [5]). With NMIBC, approximately 70–80% are stage Ta, 20% are T1, and 10% are CIS [6]. Stage Ta tumors are generally low grade, with only about 7% diagnosed as high grade [7]. Stage Ta tumors have a papillary appearance (with increased surface area) and are limited to the urothelium with no infiltration of the deeper lamina propria or underlying muscle. Stage T1 tumors show early invasiveness, crossing the basement membrane into the lamina propria, although not yet invading the deeper muscle layers. There is significant risk of understaging patients with these T1 NMIBCs especially high-grade tumors [8]. CIS (also known as Tis) is restricted to the urothelial layer, but its anaplastic morphology indicates that it is likely a precursor to the development of invasive high-grade bladder cancer. Early, low-grade lesions carry a 50–70% recurrence rate and a 10–15% risk of progression to muscle-invasive disease over a 5-year period [9, 10]. Between 40% and 83% of patients with CIS will develop muscle invasion if left untreated [11, 12]. About 30% of patients with high-grade TCC have muscle-invasive cancer at initial diagnosis, half of whom will go on to have distant metastasis within 2 years, and 60% of whom will not survive 5 years, despite aggressive treatment [8, 13, 14].

## 2. Treatments for Transitional Cell Carcinoma

 Standard therapy combines intravesical therapy with or without transurethral resection (TUR). TUR is typically the first treatment for visible lesions, although this surgery sometimes incompletely removes the tumor, necessitating a second TUR [15, 16]. For patients at low risk of tumor recurrence (and without a bladder wall puncture), early instillation of a chemotherapeutic agent following TUR is now the standard treatment recommendation. Intravesical chemotherapy, however, is not without risk given that the urothelium is already potentially destabilized by the field effect of carcinogen exposure [17]. Mitomycin C, epirubicin, and doxorubicin have all been determined to be valuable options [18]. High-grade Ta, T1, or CIS tumors put patients at an increased risk for recurrence and, more significantly, progression. Recommended treatment for patients with these high-grade tumors is TUR followed by intravesical treatment with the immunotherapeutic agent Bacillus Calmette-Guérin (BCG) and maintenance immunotherapy for at least 1 year [19, 20].

In patients whose cancer fails to respond to these bladder-sparing treatments and who refuse surgery or are not suitable patients for surgery, the treatment choices become limited. Patients with NMIBC recurrence after intravesical chemotherapy can benefit from BCG instillations [21, 22]. However, if this treatment fails, the treatment options are restricted and comprise a modified immunotherapy treatment, low-dose BCG plus interferon-alpha [23], chemotherapy with intravesical gemcitabine [24, 25] or docetaxel [26]. Cystectomy, however, remains the standard treatment for high-risk patients whose cancers have been unsuccessful with BCG therapy and/or chemotherapy [27]. Patients who receive a cystectomy before their bladder cancer progresses to a muscle invasive disease have shown an excellent disease-free survival [28]. However, cystectomy is not without the possibility of mortality and significant morbidity, especially in the older patient with associated comorbidities [29].

NMIBC that fails BCG is in need of other bladder-sparing treatment options. Here, we will evaluate the potential for the use of oncolytic viruses in the treatment of bladder cancers and try to make a case as to why further clinical evaluation should be pursued.

## 3. Transitional Cell Carcinoma as a Target for Oncolytic Viruses

Oncolytic virus therapy exploits the altered environment in the tumor cell, allowing the viruses to replicate in and lyse tumor cells, but not normal cells (reviewed in [30–32]). Many different viruses have been examined in preclinical studies for oncolytic properties with several moving into early phase clinical trials. The urinary bladder is an excellent organ to evaluate local oncolytic viral therapy for a number of reasons: (1) the urethra permits easy intravesical instillation allowing the tumor to be exposed to large titers of vector [33]; (2) the bladder is an isolated organ and the trilaminar (asymmetric) unit membrane limits systemic exposure [34–36]; (3) the success of BCG therapy has shown the immunosensitivity of bladder cancer providing a basis for examination of other immunomodulatory agents for therapy [37]; (4) the papillary configuration of NMIBC increases surface area for topical application; (5) there is an urgent need for more bladder-sparing therapies for patients failing conventional therapies.

## 4. Adenovirus (Ad) as an Oncolytic Agent

 Ad is a nonenveloped, linear, double-stranded DNA virus with a genome of approximately 36 kb. The human Ad subgroup C, which contains 2 of the most studied serotypes (types 2 and 5), is widespread in the population and associated with a mild upper respiratory tract infection. Wild-type Ads have been genetically modified to take advantage of the altered tumor environment to allow selective replication. Two general approaches have been used to generate this tumor selectivity. The first is to delete gene functions that are critical for efficient viral replication in normal cells but are expendable in tumor cells [38, 39]. ONYX-015 (dl1520 or CI-1042) was the first conditionally replication-competent engineered Ad to enter a clinical trial. It contains a deletion of the E1B-55 kDa gene and demonstrated oncolytic activity in cancer cells with mutant p53, but only limited cytotoxicity in normal human cells with wild-type p53 function [40, 41] (however, it has become clear that this is not the reason for selective replication) [42]. A second general approach is to limit the expression of the E1A gene product through the use of tumor- and/or tissue-specific promoters [43, 44]. E1A functions to stimulate S phase and transcriptional activation of both cellular and viral genes, allowing virus replication to proceed. An example is the CN706 virus in which the E1A gene is transcriptionally controlled by the PSA promoter, resulting in a virus that selectively replicates in tissue with high PSA levels [45]. There are many other examples of selectively replicating oncolytic Ads that have been reviewed elsewhere [46, 47].

Ramesh et al. have recently reported both preclinical and clinical results of their oncolytic Ad, CG0070 for the treatment of bladder cancer [48]. CG0070 is a selectively replicating Ad in which the human E2F-1 promoter drives expression of the E1A viral gene. E2F-1 is regulated by the retinoblastoma tumor suppressor protein (Rb), which is commonly mutated in many bladder cancers [49–51]. Loss of Rb binding to E2F-1 results in a transcriptionally active E2F [52]. In addition, CG0070 encodes the human granulocyte macrophage-colony stimulating factor (GM-CSF) [53], a cytokine that stimulates the maturation and recruitment of macrophages and dendritic cells and is known to be a potent inducer of local antitumor immunity [54]. CG0070 preferentially replicates in Rb protein-defective bladder cancer cells resulting in production of GM-CSF that activates the host immune response. The tumor selectivity of CG0070 was indicated by the 100-fold higher replication and 1000-fold greater cytotoxicity in bladder TCC cells compared to normal human fibroblast cells. Expression of GM-CSF in MRC-5 (normal lung fibroblast) cells was up to 45-fold lower than in the TCC cell lines used in these experiments. CG0070 showed tumor killing in orthotopic and subcutaneous human xenograft bladder tumor models. A significant antitumor effect was seen after five intratumoral injections of CG0070 at concentrations up to 3 × 10^10^ viral particles per dose. Half of the mice (5 of 10) treated with the highest dose showed complete tumor regression compared with no regression in mice treated with PBS. GM-CSF expression might enhance the anticancer effect of CG0070 because uninfected local tumor and potentially distant tumor metastases may be targeted by the induced immune response. However, the human GM-CSF encoded by this virus is species specific; therefore, the antitumor effects seen were likely only a result of the oncolytic activity of CG0070 [55].

These promising preclinical data led to a phase I/II clinical trial with CG0070 that focused on NMIBC (CIS, Ta, and T1 groups) in patients with recurrent bladder cancer after BCG treatment [56]. Results of single and multidose (weekly 6x or monthly 3x) cohorts with CG0070 delivered intravesically into the bladder at doses up to 10^13^ virus particles in 35 patients showed a response rate of 23% in single dose and 64% in multidose groups as assessed by cystoscopy and urine cytology or biopsy. Local toxicities (dysuria, bladder pain, and frequency) and flu-like symptoms were the most common adverse events observed [57, 58]. To our knowledge, this is the first report of a clinical trial using an oncolytic Ad in bladder cancer. The encouraging results have led to a phase II/III trial that is set to begin in mid-2012 evaluating CG0070 in patients with NMIBC who have failed BCG therapy [59].

## 5. Oncolytic Herpes Simplex Virus (HSV)

**Figure 1:**
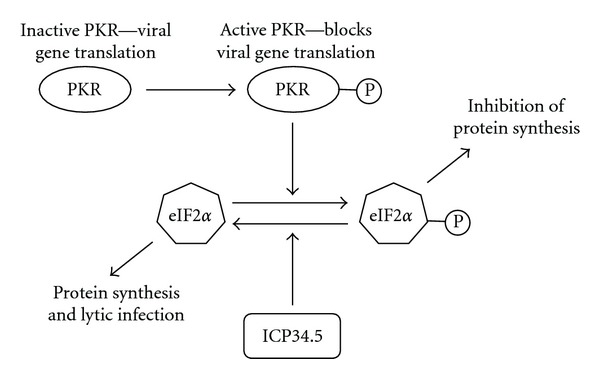
*Oncolytic mechanism of Herpes Simplex Virus.* Viral RNA activates the double-stranded-RNA- (dsRNA-) dependent protein kinase (PKR) by phosphorylation, which causes eIF2*α* phosphorylation and inhibition of translation of the viral genes. HSV *Υ*-34.5 gene encodes the ICP34.5 protein that acts to dephosphorylate eIF2*α* allowing protein synthesis to continue. In many cancer cells with activated Ras, PKR is not phosphorylated. Deletion of the *Υ*-34.5 gene from HSV results in attenuation of viral replication in normal cells but allows a lytic infection in cancer cells that have defects in this signaling pathway.

HSV is a large (150–200 nm diameter) enveloped virus [60] with a double-stranded DNA genome of approximately 150 kb [61]. HSV commonly causes infections in the orofacial region (HSV-1) and in the genital region (HSV-2) (reviewed in [62]). Multiple genetic manipulations to HSV have allowed the development of viruses that selectively replicate in cancer cells. One mutation that has been examined is the inactivation of the viral ICP6 (UL39) gene, which codes for the large subunit of ribonucleotide reductase (RR) [63, 64]. RR plays a key role in making the deoxyribonucleotides (dNTPs) that are needed for DNA synthesis [65]. The RR levels are elevated in dividing tumor cells but low in normal cells. This mutation therefore renders the virus dependent on the cellular enzyme resulting in tumor selectivity. A second modification that has been investigated is the inactivation of the *Υ*-34.5 gene that encodes the ICP34.5 protein which is important for viral replication [66], viral exit from cells [67], prevention of the early shut-off of protein synthesis [68], and neurovirulence [69] (Figure 1). In normal cells, the double-stranded-RNA-(dsRNA-) dependent protein kinase (PKR) shuts off protein synthesis and prevents viral replication [70]. Tumor cells often have defects in this signaling pathway and thus allow viral replication [71]. Mutation of the viral thymidine kinase (UL23) gene also renders the virus dependent on host cell TK expression [72].

 Oncolytic HSV armed with immunomodulating transgenes such as GM-CSF [73], interleukin-2 [74], interleukin-12 [75], and B7-1 [76] has also been developed. In addition, conditionally replicating HSV has been used to deliver gene products that convert pro-drugs into cytotoxic agents. One example of this is rRp450, a replication-selective HSV that is deleted for RR and codes for the rat cytochrome *P*450 transgene. Cytochrome *P*450 activates prodrugs such as cyclophosphamide (CPA) to generate highly toxic metabolites. It has been shown *in vitro* that rRp450 oncolytic cell killing was improved by administration of CPA [77]. HSV-1-encoded thymidine kinase (HSV-TK) phosphorylates the prodrug ganciclovir, and the resulting activated metabolite induces increased cell death compared to virus oncolysis alone. HSV-TK activation of ganciclovir in infected cells also stops viral replication [78]. HSV-TK and ganciclovir could therefore be used as a safety mechanism to prevent virus spread if serious virus toxicity were to develop.

Cozzi et al. reported on two attenuated, replication-competent HSVs, G207 and NV1020, for treatment of bladder cancer in a mouse model [79]. Both G207 and NV1020 are genetically modified oncolytic viruses based on HSV type-1 [80, 81]. G207 is modified by deletions of both copies of *Υ*-34.5 and interruption of the UL39 gene (RR) [82]. NV1020 has a deletion in the TK region of the genome and a 15 kb deletion across the junction of the long and short segments of the HSV-1 genome. Both G207 and NV1020 were compared to BCG treatment and proved very successful when delivered by *intravesical instillation* weekly for 3 weeks (10^7^ PFU). Ten of 11 animals in the control group revealed bladder tumors at autopsy. A significant increase in tumor clearance was shown in the treated groups, with tumors observed in only six of 12 animals in the BCG group, 5 of 13 animals in the G207 group, and only 2 of 12 animals in the NV1020 group. These encouraging results with oncolytic HSV in bladder cancer suggest that there should be further evaluation of intravesical oncolytic HSV therapies for bladder cancer in clinical trials.

Recently, Simpson et al. have reported results with OncoVEX^GALV/CD^ as an intravesical therapy for bladder cancer. OncoVEX^GALV/CD^ is an oncolytic HSV-1 that expresses a potent prodrug activating gene Fcy::Fur which combines the activity of the yeast cytosine deaminase (CD) and uracil phosphoribosyltransferase (UPRT) to sensitize cells to 5-fluorocytosine (5-FC) [83]. It also contains the fusogenic gibbon ape leukemia virus envelope (GALV) glycoprotein that can be used to cause an anti-tumor immune response [84]. Deletion of the viral ICP34.5 genes in OncoVEX^GALV/CD^ results in tumor selective viral replication. An 84.5% decrease in tumor size in the presence of both OncoVEX^GALV/CD^ and 5-FC when compared with control was observed in the rat AY27 orthotopic bladder tumor model.

OncoVEX^GM-CSF^ similar in structure to OncoVEX^GALV/CD^ has shown promising results in phase I and II clinical trials for a variety of cancers; including breast, head and neck, and malignant melanoma [85, 86]. It has been modified by deletion of ICP34.5 and replacement of ICP47 with the coding sequence for human GM-CSF under the control of the human cytomegalovirus immediate early promoter [87, 88]. ICP47 blocks the major histocompatibility complex (MHC) class I antigen presentation pathway by binding to the transporter associated with antigen presentation (TAP) protein [89, 90]. As a safety mechanism, the TK gene remains intact, maintaining sensitivity to antiviral agents. A phase II study of OncoVEX^GM-CSF^ in metastatic melanoma demonstrated a 26% objective response rate after direct injection into accessible melanoma lesions. Patients that showed a response had regression of both injected and noninjected lesions [81]. The safety profile of oncolytic HSVs in both the phase I and II studies has been encouraging, and further evaluation is underway with a phase III trial for unresectable stage III or IV melanoma to determine significance [91]. Multiple oncolytic mutants have shown promise in both preclinical bladder cancer models and in clinical trials for other cancers. Thus, there is a huge untapped potential for oncolytic HSV to be used in the treatment of bladder cancer patients.

## 6. Reovirus

Reoviridae are a family of viruses that includes viruses that infect the gastrointestinal tract and respiratory system. Human reoviruses contain 10 segments of double-stranded RNA and a double shell of proteins that compose the inner capsid or core and the outer capsid.

**Figure 2:**
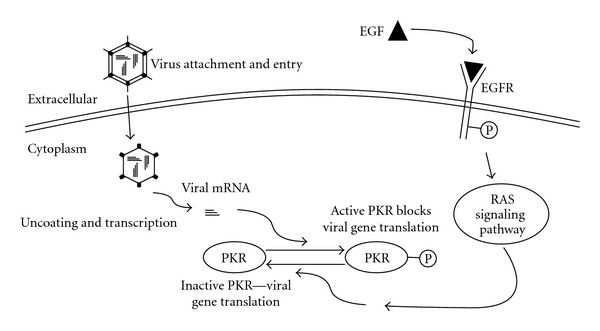
*Oncolytic mechanism of reovirus.* Similar to HSV in normal or untransformed cells, double-stranded reovirus RNA activates the double-stranded-RNA- (dsRNA-) dependent protein kinase (PKR), which causes eIF2*α* phosphorylation and inhibition of translation of the viral genes. In many cancer cells there is an activated epidermal growth factor receptor (EGFR) or mutation in downstream signaling cascades such as Ras. This dysregulated growth factor signaling means PKR is not phosphorylated thus allowing translation of viral genes and a productive lytic infection that results in cell lysis.

 The first report of the oncolytic properties of these viruses came from the realization that the virus replicated in transformed cell lines but not in normal cells [92]. Since then it has been confirmed that reovirus oncolysis requires overexpression of the Ras-signaling cascade in target cells or upregulated growth factor signaling [93, 94]. In normal cells, reovirus (double-stranded RNA) activates the double-stranded RNA-dependent protein kinase (PKR) and blocks viral protein translation by inhibiting the eukaryotic initiation factor 2*α* (eIF2*α*) [95]. In cancer cells with activated Ras, reovirus-activated protein kinase activation is inhibited, allowing viral protein synthesis and an oncolytic infection to occur (Figure 2). Around 30% of all cancers have a mutation in the Ras protein [96]. The majority of the remaining cancers still rely on some form of mutation in the epidermal growth factor (EGF) pathway. This can occur through mutation of other downstream elements or from growth factor ligand/receptor interactions that initiate Ras function. Mutated receptor tyrosine kinase proteins that are constitutively active can also occur [97]. Up to 90% of TCC have an overactive EGF pathway [98].

 Hanel et al. demonstrated oncolytic activity of reovirus *in vitro* and in an orthotopic bladder tumor model [99]. Female rats were treated twice a week for 3 weeks with low, medium, and high doses (2.5 × 10^5^, 2.5 × 10^6^, 2.5 × 10^7  ^ PFU) of intravesical reovirus or BCG as control. Complete tumor response was observed in 90% at 100 days after tumor implantation in medium- and high-dose reovirus-instilled animals, while the highest survival in the BCG-treated groups was 50%. Despite these encouraging results, little research has gone into further use of reovirus for bladder cancer.

 In the first-in-man study, patients with a variety of malignancies received escalating doses of intratumoral reovirus at levels ranging from a single injection of 10^7^ PFU to three injections of 10^10^ PFU. The main symptoms were headaches and a flu-like illness [100]. Since then, multiple phase I and II studies have been completed. A phase I dose escalation study was performed on 12 patients with recurrent gliomas, evaluating an unmodified reovirus administered through intratumoral injection. A maximum tolerated dose was not reached, and treatment was well tolerated [101]. A phase II study was also performed with i.v. administration of wild-type reovirus in patients with bone and soft tissue sarcomas that had metastasized to the lung [102]. These clinical studies show that both intratumoral and i.v. administration of wild-type reovirus in patients was safe and well tolerated. These early clinical results, as well as the relatively low risk due to reovirus' limited pathogenicity in humans, highlight the promising potential for this oncolytic agent to expand its clinical potential to include bladder cancer.

## 7. Oncolytic Vaccinia Virus (VAC)

VAC has a large (~200 kb) linear double-stranded DNA genome that replicates exclusively in the cytoplasm. VAC infects many different cell types with high efficiency. VAC encodes many of the proteins required for robust virus replication in normal cells (reviewed in [103–105]).

**Figure 3:**
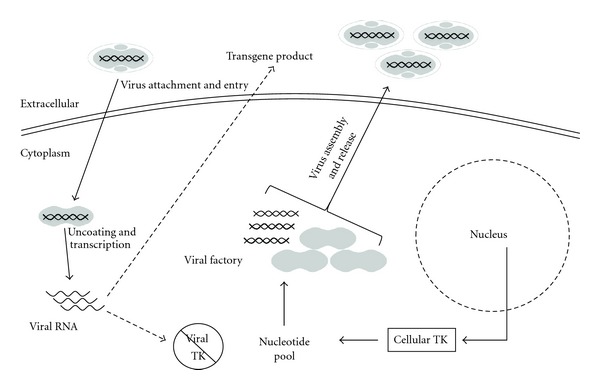
*Tumor selectivity of oncolytic vaccinia viruses.* In normal cells, the wild-type virus encodes a range of gene products that allow virus replication in the cytoplasm of host cells. These products include, but are not limited to, thymidine kinase (TK) and ribonucleotide reductase (not shown), which generate a nucleotide pool to facilitate virus replication. In normal cells, viruses deleted of these essential genes are unable to undergo productive replication. However, in tumor cells, mutations cause dysregulation of numerous pathways, including pathways that allow for unchecked proliferation. One result of these unregulated proliferative pathways is a high level of production of nucleotides, creating a favorable environment for vaccinia virus replication. The mutated viruses are able to replicate, express transgenes (if present), and lyse tumor cells.

 In recent years, there has been extensive research into VAC as a cancer therapy. Genetic mutations that occur in cancer can generate an environment that is optimal for VAC replication; thus, some of the viral genes involved in replication become expendable. Therefore, deletion of these genes from the viral genome greatly reduces the ability of the virus to replicate productively in most normal cells, while allowing them to retain their replication ability in cancer cells. A range of VAC gene deletions with such properties has been investigated as a means to increase tumor selectivity of the virus. Oncolytic VACs reported to date are most commonly generated by mutations that inactivate J2R (thymidine kinase, TK) and C11L/R (vaccinia growth factor, VGF), which reduce virulence in the host (animals) and favors virus growth in rapidly dividing cells [106, 107]. Cellular TK is briefly expressed during S phase in normal cells but is constitutively expressed at high levels in a large number of cancers throughout the cell cycle (Figure 3) [108]. VGF is an EGF homologue that can bind to cellular EGF receptor [109, 110]. VGF is released from infected cells to induce proliferation, and VAC strains with VGF deletions show selective replication in cancers with an activated EGFR. The VGF deletion can be combined with the TK deletion to generate a further attenuated virus [111]. Recently, Gammon et al. have shown that, by deleting the gene encoding the small subunit of VAC RR (F4L), one can render the virus highly dependent upon the cellular homolog to provide the complementing activity that is needed for virus replication [112]. The F4L deleted viruses are thus quite highly attenuated in infected animals and show a tropism that greatly favors cells containing high levels of RR. This virus may be specifically useful for treating recurrent NMIBC where patients have failed BCG and RR-targeted chemotherapies such as gemcitabine.

 Oncolytic VACs armed with a variety of transgenes have also generated much attention recently. Viruses have been developed that encode cytokines such as GM-CSF [113] and interferon-beta (IFN-*β*) [114]. Interestingly, VAC encodes an inhibitor of type-I IFNs, the B18R gene product. An oncolytic VAC has been constructed with deletion of the B18R gene and insertion of the INF-*β* gene. VAC replication should be highly restricted in normal cells, but permissive in IFN-resistant cancer cells. Furthermore, IFN-*β* is predicted to elicit an increased anti-cancer response [115]. Anti-angiogenic agents have been expressed to help complement the oncolytic effects of the virus [116]. Finally, prodrug-converting enzymes have been introduced into VACs to convert nontoxic prodrugs into toxic products within the tumor [117].

 Gomella et al. reported a phase I study where increasing doses of intravesical wild-type VAC (the Dryvax vaccine) were administered to patients with muscle-invasive-bladder carcinoma for whom radical cystectomy was planned as final treatment [118]. The study examined 4 patients that were treated 3 times over 2 weeks with a maximum dose of 10^8^ PFU prior to cystectomy. This study demonstrated that even wild-type VAC can be administered safely into the bladder and cause the recruitment of lymphocytes and induction of a local inflammatory response. Besides mild local toxicity, no serious treatment-related side effects were reported. The excellent patient tolerance of intravesical VAC and the significant immune infiltrates seen after instillation support the potential use of VAC as an oncolytic agent for intravesical bladder cancer therapy.

 Clinical data have now been published for the first targeted and armed oncolytic poxvirus to be used in the clinic, JX-594. It is a Wyeth strain VAC with inactivation of J2R (viral TK) and insertion of the GM-CSF gene [119]. Phase I clinical data reported on the intratumoral injection of seven patients with surgically incurable cutaneous melanoma [120]. Multiple injections with JX-594 at doses up to 2 × 10^7^  PFU/lesion were given over 6 weeks. Overall the treatment had controlled side effects that included transient flu-like symptoms and local inflammation, with the occasional pustule formation at the site of injection. Five of seven patients had some response to the treatment with one patient having a complete remission. In another phase I trial, direct injection of JX-594 into liver tumors was well tolerated, with virus replication, expression of active GM-CSF, and tumor killing observed [121]. In this dose escalation study, patients who had previously received multiple therapies were injected with up to 3 × 10^9^ PFU every 3 weeks with an average of 3.4 treatments. Of the ten patients assessed, three showed partial responses, six had stable disease, and one showed progression. JX-594 was generally well tolerated up to the maximum tolerated dose of 10^9^ PFU. The dose-limiting toxicity, hyperbilirubinemia, was seen at 3 × 10^9^ PFU because of tumor swelling, causing a bile-duct obstruction.

 Partial results of a phase II trial examining intratumoral administration JX-594 in patients with hepatocellular carcinoma have been reported. They reported that 6-month survival of patients treated with low-dose (10^8^ PFU) was 48%, and with high-dose (10^9^ PFU) was 75%. The 12-month survival was 18% and 75%, respectively [122]. Efficient tumor killing seems to be a dose-dependent property that can be limited by toxicity following systemic or hepatic delivery. JX-594 has recently been tested in a phase I dose-escalation trial through i.v. administration in 23 cancer patients with advanced solid tumors that had developed resistance to multiple other treatments. This study established a maximum feasible dose of 3 × 10^7^ PFU/Kg (equivalent to a total dose of about 2 × 10^9^ PFU) [123]. This is the first report of replication and transgene expression in metastatic tumors after i.v. administration of an oncolytic virus. Because of the anatomical isolation of the bladder, it may be possible to administer higher doses of virus locally without systemic effects. Encouraging clinical results for the treatment of other cancers with oncolytic VAC further suggest that the investigation of oncolytic VAC as a bladder cancer therapy should be a priority.

## 8. Conclusion

 Surgery, BCG, and chemotherapy dramatically slow the progress of bladder cancer but do not eradicate the disease totally. Patients with NMIBC that fail BCG therapy are in need of other bladder-sparing treatment options. This paper discussed the potential of oncolytic viruses as a treatment option in bladder cancer. Encouraging safety profiles and antitumor activity have been demonstrated with a variety of oncolytic viruses. However, very little preclinical, let alone clinical, data have been reported for oncolytic viruses in bladder cancer.

 Although the agents described in this paper have shown convincing preclinical and early clinical results, the ultimate proof of antitumor efficacy and safety still need to be provided by randomized phase III clinical trials. Therefore, there remains uncertainty in their ability to have significantly better effects over current therapies in patients. As with any viral therapy, the main obstacle to overcome is delivery of sufficient virus particles to the target tissue in order to have a desired therapeutic effect. However, the bladder may provide an environment to help overcome some of these issues. Local delivery to the isolated environment of the bladder can allow the tumor to be exposed to large titers of virus with limited systemic exposure and consequent toxicity. The papillary configuration of the NMIBC lends itself to topical application of agents with tropism to urothelial cancer. Furthermore, these agents appear to be noncarcinogenic like BCG and but unlike BCG could potentially be administered earlier in the course of therapy (immediately after TUR) without the significant risk of severe systemic illness. In addition, direct oncolysis by selective replication in transformed NMIBC cells could potentially avoid inflammation and the profound symptoms of cystitis. Combinations of these viral agents targeting multiple or sequential pathways could prevent the development of resistance, with little added toxicity. Thus, the potential high degree of safety and efficacy predicted for oncolytic virus therapy of urothelial cancer warrants immediate further investigation at both the preclinical and clinical levels.
